# Supplementary material for: Prognostic prediction models for clinical outcomes in patients diagnosed with visceral leishmaniasis: protocol for a systematic review
Source: BMJ Open. 2023 Oct 25;13(10):e075597. doi: 10.1136/bmjopen-2023-075597 (PMC10603465; doi:10.1136/bmjopen-2023-075597)
Supplement: Supplementary data [file bmjopen-2023-075597supp002.pdf]

Supplemental Material - Search strategy

Tables 1-5 describe the search terms for each database (Ovid MEDLINE; Ovid Embase, the Web of Science Core Collection, SciELO and LILACS, respectively). All searches performed on 1st March 2023. No limit on publication date (from database inception to 1st March 2023).

Subsequent Google scholar grey literature review: ("visceral leishmaniasis" OR "Kala-azar") AND ("model" OR "prediction" OR "score" OR "prognostic"), run on July 17th 2023. The first 200 results were reviewed for relevance.

Query #	Query terms
1	Leishmaniasis, Visceral/
2	((Leishmaniasis and Visceral) or (Leishmania and infantum) or (Leishmania and donovani) or (Kala and azar)).ti,ab,kw.
3	1 or 2
4	Validat$.ti,ab. or Predict$.ti. or Rule$.ti,ab. or (Predict$ adj2 (Outcome$ or Risk$ or Model$)).ti,ab. or ((History or Variable$ or Criteria or Scor$ or Characteristic$ or Finding$ or Factor$) adj2 (Predict$ or Model$ or Decision$ or Identif$ or Prognos$)).ti,ab. or (Decision$ adj2 (Model$ or Clinical$)).ti,ab. or (Prognostic adj2 (History or Variable$ or Criteria or Scor$ or Characteristic$ or Finding$ or Factor$ or Model$)).ti,ab.
5	logistic models/
6	decision*.ti,ab
7	5 and 6
8	4 or 7
9	(Stratification or ROC Curve or Discrimination or Discriminate or c-statistic or c statistic or Area under the curve or AUC or Calibration or Indices or Algorithm or Multivariable).ti,ab.
10	roc curve/
11	8 or 9 or 10
12	3 and 11

Table 1: Search strategy - Ovid MEDLINE

**Query #**	**Query terms**
1	visceral leishmaniasis/
2	((Leishmaniasis and Visceral) or (Leishmania and infantum) or (Leishmania and donovani) or (Kala and azar)).ti,ab,kw.
3	1 or 2

4	Validat$.ti,ab. or Predict$.ti. or Rule$.ti,ab. or (Predict$ adj2 (Outcome$ or Risk$ or Model$)).ti,ab. or ((History or Variable$ or Criteria or Scor$ or Characteristic$ or Finding$ or Factor$) adj2 (Predict$ or Model$ or Decision$ or Identif$ or Prognos$)).ti,ab. or (Decision$ adj2 (Model$ or Clinical$)).ti,ab. or (Prognostic adj2 (History or Variable$ or Criteria or Scor$ or Characteristic$ or Finding$ or Factor$ or Model$)).ti,ab.
5	statistical model/
6	decision*.ti,ab.
7	5 and 6
8	4 or 7
9	(Stratification or ROC Curve or Discrimination or Discriminate or c-statistic or c statistic or Area under the curve or AUC or Calibration or Indices or Algorithm or Multivariable).ti,ab.
10	receiver operating characteristic/
11	8 or 9 or 10
12	3 and 11

Table 2: Search strategy - Ovid Embase

Query #	Query terms
1	TS=((Leishmaniasis and Visceral) or (Leishmania and infantum) or (Leishmania and donovani) or (Kala and azar))
2	TS=(Validat$ or Rule$ or (Predict$ near/2 (Outcome$ or Risk$ or Model$)) or ((History or Variable$ or Criteria or Scor$ or Characteristic$ or Finding$ or Factor$) near/2 (Predict$ or Model$ or Decision$ or Identif$ or Prognos$)) or (Decision$ near/2 (Model$ or Clinical$)) or (Prognostic near/2 (History or Variable$ or Criteria or Scor$ or Characteristic$ or Finding$ or Factor$ or Model$)))
3	TI=(Predict$)
4	TS=(Stratification or ROC Curve or Discrimination or Discriminate or c-statistic or c statistic or Area under the curve or AUC or Calibration or Indices or Algorithm or Multivariable)
5	#4 OR #3 OR #2
6	#5 AND #1

Table 3: Search strategy - Web of Science Core Collection

**Query #**	**Query terms**
1	All indexes: ((Leishmaniasis and Visceral) or (Leishmania and infantum) or (Leishmania and donovani) or (Kala and azar))

2	All indexes: Validat* or Rule* or (Predict* and (Outcome* or Risk* or Model*)) or ((History or Variable* or Criteria or Scor* or Characteristic* or Finding* or Factor*) and (Predict* or Model* or Decision* or Identif* or Prognos*)) or (Decision* and (Model* or Clinical*)) or (Prognostic and (History or Variable* or Criteria or Scor* or Characteristic* or Finding* or Factor* or Model*)) or Stratification or ROC Curve or Discrimination or Discriminate or c-statistic or c statistic or Area under the curve or AUC or Calibration or Indices or Algorithm or Multivariable
3	1 AND 2

Table 4: Search strategy – SciELO

Query #	Query terms
1	(tw:(((Leishmaniasis and Visceral) or (Leishmania and infantum) or (Leishmania and donovani) or (Kala and azar)))) AND (tw:(Validat* or Rule* or (Predict* and (Outcome* or Risk* or Model*)) or ((History or Variable* or Criteria or Scor* or Characteristic* or Finding* or Factor*) and (Predict* or Model* or Decision* or Identif* or Prognos*)) or (Decision* and (Model* or Clinical*)) or (Prognostic and (History or Variable* or Criteria or Scor* or Characteristic* or Finding* or Factor* or Model*)) or Stratification or ROC Curve or Discrimination or Discriminate or c-statistic or c statistic or Area under the curve or AUC or Calibration or Indices or Algorithm or Multivariable))

Table 5: Search strategy - LILACS
