# Supplementary material for: Prognostic prediction models for clinical outcomes in patients diagnosed with visceral leishmaniasis: protocol for a systematic review
Source: BMJ Open. 2023 Oct 25;13(10):e075597. doi: 10.1136/bmjopen-2023-075597 (PMC10603465; doi:10.1136/bmjopen-2023-075597)
Supplement: Author's manuscript [file bmjopen-2023-075597.draft_revisions.pdf]

BMJ Open

BMJ Open is committed to open peer review. As part of this commitment we make the peer review history of every article we publish publicly available.

When an article is published we post the peer reviewers' comments and the authors' responses online. We also post the versions of the paper that were used during peer review. These are the versions that the peer review comments apply to.

The versions of the paper that follow are the versions that were submitted during the peer review process. They are not the versions of record or the final published versions. They should not be cited or distributed as the published version of this manuscript.

BMJ Open is an open access journal and the full, final, typeset and author-corrected version of record of the manuscript is available on our site with no access controls, subscription charges or pay-per-view fees (http://bmjopen.bmj.com).

If you have any questions on BMJ Open's open peer review process please email info.bmjopen@bmj.com

BMJ Open

Protocol for a Systematic Review of Prognostic Prediction Models describing Clinical Outcomes in Patients Diagnosed with Visceral Leishmaniasis

Journal:	*BMJ Open*
Manuscript ID	bmjopen-2023-075597
Article Type:	Protocol
Date Submitted by the Author:	12-May-2023

Complete List of Authors:	Wilson, James; Infectious Diseases Data Observatory (IDDO); University of Oxford Centre for Tropical Medicine and Global Health<br>Chowdhury, Forhad; Infectious Diseases Data Observatory (IDDO); University of Oxford Centre for Tropical Medicine and Global Health<br>Hassan, Shermarke; Infectious Diseases Data Observatory (IDDO); University of Oxford Centre for Tropical Medicine and Global Health<br>Harriss, Elinor; Bodleian Health Care Libraries, University of Oxford<br>Alves, Fabiana; Drugs for Neglected Disease Initiative<br>Dahal, Prabin; Infectious Diseases Data Observatory (IDDO); University of Oxford Centre for Tropical Medicine and Global Health<br>Stepniewska, Kasia; Infectious Diseases Data Observatory (IDDO); University of Oxford Centre for Tropical Medicine and Global Health<br>Guérin, Philippe J.; Infectious Diseases Data Observatory (IDDO); University of Oxford Centre for Tropical Medicine and Global Health

Keywords:	Prognosis, Neglected Diseases, INFECTIOUS DISEASES, Systematic Review

SCHOLARONE™
Manuscripts

Protocol for a Systematic Review of Prognostic Prediction Models describing Clinical Outcomes in Patients Diagnosed with Visceral Leishmaniasis

James P Wilson[1,2†], Forhad Chowdhury[1,2], Shermarke Hassan[1,2], Eli Harriss[3], Fabiana Alves[4], Prabin Dahal[1,2], Kasia Stepniewska[1,2], Philippe J Guérin[1,2]

ABSTRACT

Introduction

Visceral leishmaniasis is a neglected tropical disease responsible for many thousands of preventable deaths each year. Affected people often struggle to access effective treatment, without which death is the norm. Risk prediction tools support clinical teams and policymakers in identifying high-risk patients who could benefit from more intensive management pathways. Investigators interested in using their clinical data for prognostic research should first identify currently available models that are candidates for validation and possible updating. Addressing these needs, we aim to identify, summarise and appraise the available models predicting clinical outcomes in patients diagnosed with visceral leishmaniasis.

Methods and analysis

We define study eligibility using the PICOTS (population, index model, comparator model, timing, setting) framework. Data extraction, appraisal and reporting will follow current methodological guidelines. We will search five bibliographic databases using terms developed for the identification of prediction models. Screening, data extraction, and risk of bias assessment will be performed in duplicate. Discordance will be resolved by a third independent reviewer. We will use the PROBAST tool to assess risk of bias.

A narrative review will summarise our findings. Tables and figures will compare and contrast key model information, including source data, participants, model development and performance measures, and risk of bias. We will consider the strengths, limitations and clinical applicability of the identified models.

Ethics and dissemination

Ethics approval is not required for this review. The systematic review and all accompanying data will be submitted to an open-access journal. Findings will also be disseminated through conference presentations, the research group's website (www.iddo.org) and social media channels.

Registration details

This protocol has been registered with PROSPERO (ID: CRD42023417226).

Keywords

Leishmaniasis, Visceral; Review, Systematic; Clinical Decision Rules; Prognosis; Prognostic Factors;

Strengths and limitations of this study

- Even with access to treatment, mortality rates are high in patients with visceral leishmaniasis; the use of a prognostic prediction model can identify those at high-risk, informing clinicians and policymakers on appropriate case management and the optimal distribution of constrained resources.
- We present the protocol for the first systematic review that aims to identify, summarise and appraise the available prognostic models predicting clinical outcomes in people diagnosed with visceral leishmaniasis.

For peer review only

- We use a comprehensive and evidence-based search strategy to identify a broad range of prognostic models across five large bibliographic databases, with no limitations on language, publication date or treatment setting.
- Data extraction, appraisal and reporting guidelines are followed to ensure transparency and reproducibility.
- The review's scope does not include prognostic factor studies, diagnostic prediction studies or non-peer reviewed publications.

INTRODUCTION

Visceral leishmaniasis (VL), a parasitic infection transmitted between mammalian hosts via the bite of an infected sand fly, is a neglected tropical disease that disproportionately touches vulnerable people affected by forced migration, malnutrition and poverty[1] The disease often presents insidiously with fever, splenomegaly and weight-loss, and is almost always fatal without effective treatment[2] The World Health Organization (WHO) estimates an incidence of 50,000 to 90,000 cases per year resulting in approximately 400,000 disability-adjusted life years lost and over 5,000 deaths.[3,4] However, accurate estimates of disease burden are obfuscated by limited country-level reporting, evolving dense foci of infection in remote areas, and a paucity of active surveillance[1] Despite progress made over the last 20 years, successful treatment remains challenged by high drug costs, prolonged treatment courses requiring hospitalisation and frequent drug side effects[5]Patients with previous treatment failure or immunosuppressive comorbidities such as advanced human immunodeficiency virus (HIV) suffer from particularly high relapse and mortality rates.[1,6]

To optimise individual patient care and effectively balance the distribution of constrained resources, identification of patients at high risk of treatment failure and subsequent death is crucial. Risk stratification is also important on a population level; elimination programmes in endemic areas can use risk prediction tools to strategically target patients prone to treatment failure, and hence reduce the infectious reservoir driving onward transmission. Prognostic prediction models (referred henceforth as prognostic models) play a central role in VL risk stratification; informing healthcare providers, policymakers and patients on the treatment setting, treatment regimen and intensity of follow-up.[7–9]

Systematic reviews of prognostic models have been published across a range of infectious diseases,[10–12] serving not only to inform healthcare providers on available risk stratification tools, but also as a research tool to identify candidate models for external validation or updating (recalibration) using data from new settings. Indeed, the lack of external validation studies is considered the greatest barrier to the broader acceptance of prognostic models as a reliable and acceptable clinical tool.[13–15]

Aim

We will perform a systematic literature review to identify, summarise, and appraise prognostic models in patients diagnosed with VL. Specifically, we focus on models that predict clinical outcomes such as treatment failure (initial failure or relapse) and death, developed at the time of, or subsequent to diagnosis.

The review will serve two principal purposes;

For peer review only

(i) inform stakeholders, such as policymakers and clinical teams directly involved in the treatment of VL patients, on the available prognostic models and their setting-specific clinical utility, strengths and limitations.
(ii) Inform researchers interested in using their own data for the development, validation and/or updating of VL prognostic models.

METHODS AND ANALYSIS

This systematic review will adhere to PRISMA (preferred reporting items for systematic reviews and meta-analyses)[16] a reporting guideline for systematic reviews, and TRIPOD-SRMA (transparent reporting of multivariable prediction models for individual prognosis or diagnosis: checklist for systematic reviews and meta-analysis)[17] a reporting guideline for systematic reviews of prediction models.

This protocol is registered with PROSPERO (ID: CRD42023417226). We used the PRISMA-P checklist when writing our report.[18]

Study eligibility

We follow a PICOTS (Population, Index model, Comparator model, Outcomes, Timing, Setting)[19,20] approach to frame our review question and inclusion criteria (Table 1).

Our population of interest includes all patients with a confirmed or suspected diagnosis of VL as reported by the study authors. We include all published, peer-reviewed studies that develop, externally validate, update, or any combination thereof, a prognostic model with the intention of predicting individual clinical outcomes following VL diagnosis. To provide a broad overview of available prognostic models we include all clinical outcomes. Furthermore, we impose no limitation on the model setting or prediction horizon (elapsed time period between the intended time of model use and the outcome being predicted).

Table 1: We use a PICOTS approach to frame our research question

**Population**	All patients with a confirmed or suspected diagnosis of visceral leishmaniasis as per study authors.
**Index model**	All published prognostic models that develop, validate and/or update (recalibrate/extend) a risk model.
**Comparator model**	Not applicable.
**Outcomes**	Any clinical outcome that occurs following diagnosis.
**Timing**	All prognostic models developed at the time of, or following diagnosis. No restriction on the prediction horizon.
**Setting**	No restriction.

In accordance with expert guidance on the methodology of prediction model research[14,21,22] we define a prognostic model as a multivariable model (including 2 or more predictors) where the intention is to predict outcomes at the individual patient level. Prognostic model studies are distinguished from predictor finding or prognostic factor studies, where the intent is to investigate the effect of a single or group of factors on an outcome of interest.[23] We therefore exclude all studies that present models where the aim is not to predict risk at the individual patient level. We also exclude unpublished studies, studies that only report diagnostic prediction models, and animal studies.

For peer review only

To complement the systematic review, using the same search strategy we will identify (i) systematic reviews and meta-analyses of prognostic models, and (ii) any impact studies that investigate the clinical outcomes of using versus not using an eligible prognostic model. These studies will not be subject to formal data extraction, but will contribute to the narrative review.

Search strategy

An information specialist (EH) created the search strategies to retrieve relevant records from the following databases: Ovid Embase; Ovid MEDLINE; the Web of Science Core Collection, SciELO and LILACS. The databases were searched on 1st March 2023. The search strategy used text words and relevant indexing terms to retrieve studies describing eligible prognostic models. The Ingui search filter was combined with an additional search string developed by Geersing et al,[24,25] and adapted for Ovid Embase (Table 2) and remaining bibliographic databases for this systematic review (Supplemental Material).

Table 2: Search strategy initially developed in Ovid Embase and searched on 1st March 2023. Search queries were subsequently adapted to other bibliographic databases (Supplemental Material).

Query #	Query terms
1	visceral leishmaniasis/
2	((Leishmaniasis and Visceral) or (Leishmania and infantum) or (Leishmania and donovani) or (Kala and azar)).ti,ab,kw.
3	1 or 2
4	Validat$.ti,ab. or Predict$.ti. or Rule$.ti,ab. or (Predict$ adj2 (Outcome$ or Risk$ or Model$)).ti,ab. or ((History or Variable$ or Criteria or Scor$ or Characteristic$ or Finding$ or Factor$) adj2 (Predict$ or Model$ or Decision$ or Identif$ or Prognos$)).ti,ab. or (Decision$ adj2 (Model$ or Clinical$)).ti,ab. or (Prognostic adj2 (History or Variable$ or Criteria or Scor$ or Characteristic$ or Finding$ or Factor$ or Model$)).ti,ab.
5	statistical model/
6	decision*.ti,ab.
7	5 and 6
8	4 or 7
9	(Stratification or ROC Curve or Discrimination or Discriminate or c-statistic or c statistic or Area under the curve or AUC or Calibration or Indices or Algorithm or Multivariable).ti,ab.
10	receiver operating characteristic/
11	8 or 9 or 10
12	3 and 11

Selection process and data extraction

All references were exported to Covidence for deduplication and screening (Covidence systematic review software, Veritas Health Innovation, Melbourne, Australia. Available at www.covidence.org).[26] Google Scholar will be used as a grey literature source once screening is complete to identify additional relevant studies. The reference lists of all included studies, incorporating any identified systematic reviews, meta-analyses and impact studies, will be assessed for eligible studies.

Studies identified from the search strategy are being independently screened by two reviewers (JW, FC). Preliminary screening is at title and abstract level followed by full-text screening. A third reviewer (PD), a statistician with expertise in prediction modelling, will make the final judgement on study inclusion if discordance remains following discussion between the two screening reviewers. A flow diagram will be presented as per the PRISMA 2020 checklist.[16]

Study information will be extracted, collected and managed using the REDCap electronic data capture tools hosted at the University of Oxford[27] A data extraction form will be created based on

For peer review only

the CHARMS (critical appraisal and data extraction for systematic reviews of prediction modelling studies)[20] checklist for data extraction and PROBAST (prediction model risk of bias assessment tool; https://www.probast.org/)[22,28] for assessing risk of bias (Table 3). A pilot form will be trialled prior to formal data extraction. Two independent reviewers (JW, SH), both with expertise in prediction modelling, will independently extract the study information. Where discordance remains after discussion, a final decision will be made by a third expert reviewer (PD). Study authors will not be contacted in the event of missing information for extraction.

Table 3: Information for data extraction and subsequent summary and appraisal. Adapted from the CHARMS Checklist and PROBAST risk of bias tool. [20,22,28] *VL: visceral leishmaniasis, HIV: human immunodeficiency virus*

Domain	Key Items
Source of data	Source of data (e.g., cohort, case-control, randomised trial participants, registry data, etc.)
Participants	Participant eligibility and recruitment method (e.g., location, number of centres, setting, inclusion and exclusion criteria)
	Participant description (age, sex, primary VL or relapse case, co-morbidities including HIV co-infection)
	Details of treatments received
	How VL diagnosis is defined (whether consistent for all participants, using serology and/or microscopy, molecular testing, clinical history and physical signs, etc)
	Study dates
Outcome(s) to be predicted	Type of outcome (e.g., single or combined endpoints)
	Definition and method for measurement of outcome (for example, is mortality disease-specific or all-cause, is cure/initial failure/relapse diagnosed based on clinical symptoms and/or diagnostic testing)
	Was the same outcome definition (and method for measurement) used in all patients?
	Time of outcome occurrence or summary of duration of follow-up
	Was the outcome assessed without knowledge of the candidate predictors (i.e., blinded)?
Candidate predictors	Number and type of predictors (e.g., demographics, patient history, physical examination, laboratory parameters, HIV status, disease characteristics, etc)
	Definition and method for measurement of candidate predictors (including whether defined and measured in a similar way for all participants)
	Timing of predictor measurement (e.g., at patient presentation, at diagnosis, at treatment initiation or otherwise)
	Handling of predictors in the modelling (e.g., continuous, linear, non-linear transformations or categorised)
Sample size	Number of participants and number of outcomes/events
	Events per candidate predictor
	Whether the authors describe a sample size calculation
Missing data	Number of participants with any missing value (including predictors and outcomes)
	Number of participants with missing data for each predictor
	Handling of missing data (e.g., complete-case analysis, imputation, or other methods)
Model development	Modelling method (e.g., logistic, survival, or other)
	Modelling assumptions satisfied
	Description of participants that were excluded from the analysis with justification
	Method for selection of predictors for inclusion in multivariable modelling (e.g., all candidate predictors, pre-selection based on unadjusted association with the outcome)
	Method for selection of predictors during multivariable modelling (e.g., full model approach, backward or forward selection) and criteria used (e.g., p-value, Akaike information criterion)
	Shrinkage of predictor weights or regression coefficients (e.g., no shrinkage, uniform shrinkage, penalized estimation)
Model performance	Calibration (calibration plot, calibration slope, Hosmer-Lemeshow test), discrimination (C-statistic, D-statistic, log-rank), and overall performance measures with confidence intervals
	Classification measures (e.g., sensitivity, specificity, predictive values, net reclassification improvement) and whether a priori cut points were used
Model evaluation	Method used for testing model performance: development dataset only (apparent performance, random split of data, resampling methods, e.g., bootstrap or cross-validation, none) or separate external validation

For peer review only

	For external validations; data source and participants to be described as per 'source of data' and 'participants' domains. Definitions and distributions (including missing data) of outcome and candidate predictors.
	In case of poor external validation, whether model was adjusted or updated (e.g., intercept recalibrated, predictor effects adjusted, or new predictors added)
Results	Final and other multivariable models presented, including predictor weights or regression coefficients, intercept, baseline survival, model performance measures (with standard errors or confidence intervals)
	Any alternative presentation of the final prediction models, e.g., sum score, nomogram, score chart, predictions for specific risk subgroups with performance
	Comparison of the definition and distribution of predictors (including missing data) for development and validation datasets
Interpretation and Discussion	Study authors' interpretation of presented models (intended use, clinical utility, etc)
	Study authors' reported strengths and limitations
Miscellaneous	Source of funding / sponsor
	Any declared conflicts of interest
	Methodological guidelines used

In line with prediction modelling guidance[22] each unique data extraction record relates to an individual model developed and/or externally validated within a published study.

Risk of bias

Risk of bias will be summarised and reported separately for each model development and external validation using PROBAST; a tool for assessing risk of bias and applicability of prediction model studies[28] Two reviewers (JW, SH), will answer 20 signalling questions across 4 domains (participants, predictors, outcome and analysis), which will be used to judge the overall risk of bias as either 'low', 'high' or 'unclear'.[22] Discordance between the two reviewers will be resolved by a third reviewer (PD).

If impact studies are identified, their methodological quality will be identified using tools such as the revised Cochrane Risk of Bias tool for randomised comparative designs[29] or the ROBINS-I (the risk of bias in non-randomised studies of interventions) tool for studies using a non-randomised comparative design[30]

Data synthesis

We will present a narrative synthesis of our review findings. Key extracted information will be summarised in tabular form. Performance statistics, including measures of calibration and discrimination, where available, will be presented alongside their derivation method (i.e., whether an apparent performance measure, from an internal validation, or from an external validation).

Figures will be used to concisely communicate important information, including (i) summary of the candidate and final predictors included in each model, and (ii) risk of bias assessment across the 4 domains (participants, predictors, outcomes and analysis).

Strengths and limitations of the identified models will be considered.

Given our aim is not to compare multiple external validations of a single model, we will not be performing a meta-analysis[31]

DISCUSSION

Visceral leishmaniasis remains a neglected tropical disease that affects some of the most disadvantaged communities in the world. Thoughtful risk stratification of patients using prognostic

For peer review only

models can assist clinical decision making and inform policy, facilitating the optimal allocation of limited resources.

However, the adoption of a successful model requires evidence of accuracy, generalisability and efficacy. Indeed, external validation is considered an essential step prior to model use.[21]

This review will not only serve healthcare providers and policymakers in identifying relevant risk stratification tools, but also provide a resource for research groups aiming to identify, validate and/or update prognostic models.[32] The Infectious Diseases Data Observatory (IDDO; www.iddo.org) is developing a data repository of individual participant data (IPD) from VL clinical trials and observational studies.[33] IPD present an exciting opportunity in their application to the development, validation and updating of prognostic models.[7]

In summary, we present a protocol for the systematic review of prognostic models of clinical outcomes for patients diagnosed with VL. With the aim of identifying, summarising and appraising the available risk models, we hope to provide a current reference to stakeholders engaged in VL patient care, policy and research.

For peer review only

Ethics and dissemination

Ethics approval is not required for this review. The systematic review will be submitted to an open-access journal for peer review and publication. Findings will also be disseminated through conference presentations, the research group's website (www.iddo.org) and social media channels. All collected data will be made freely available as supplemental material submitted during publication.

Patient and public involvement

Patients or the public were not involved in the design, or conduct, or reporting, or dissemination plans of our research.

Author affiliations

[1]Infectious Diseases Data Observatory (IDDO), Oxford, UK
[2]Centre for Tropical Medicine and Global Health, Nuffield Department of Medicine University of Oxford, Oxford, UK
[3]Bodleian Health Care Libraries, University of Oxford, Oxford, UK
[4]Drugs for Neglected Diseases initiative, Geneva, Switzerland

[†]Corresponding author

Twitter

The Infectious Diseases Data Observatory: @IDDOnews
James P Wilson: @jamesemaj

Emails

James P Wilson: james.wilson@iddo.org
Forhad Chowdhury: forhad.chowdhury@ndm.ox.ac.uk
Shermarke Hassan: shermarke.hassan@iddo.org
Eli Harriss: eli.harriss@bodleian.ox.ac.uk
Fabiana Alves: falves@dndi.org

Prabin Dahal: prabin.dahal@iddo.org
Kasia Stepniewska: kasia.stepniewska@iddo.org
Philippe J Guérin: philippe.guerin@iddo.org

Contributions

The study concept and design was conceived by JW, PG, KS and PD. ES developed the search strategy. JW, FC and PD will complete the literature screening. JW, SH and PD will perform data extraction. KS and PD have provided statistical support. The first draft of this manuscript was prepared by JW, who will also draft the completed systematic review. All authors critically revised and approved the submitted version.

Funding statement

JW is undertaking a PhD supported in part by the University of Oxford through the Clarendon Scholarship (https://www.ox.ac.uk/clarendon) and the Infectious Diseases Data Observatory (www.iddo.org), which is itself supported by a diverse portfolio of grants, including from the Bill & Melinda Gates Foundation (recipient: Prof. Philippe Guerin; ref: INV-004713). The funders have no role in the conception, design or conduct of the systematic review.

Competing interests statement

The authors declare no competing interests.

Word Count: 1,657

REFERENCES

1 Burza S, Croft SL, Boelaert M. Leishmaniasis. *Lancet* 2018;**392**:951–70. doi:10.1016/S0140-6736(18)31204-2

2 Guerin PJ, Olliaro P, Sundar S, *et al.* Visceral leishmaniasis: Current status of control, diagnosis, and treatment, and a proposed research and development agenda. *Lancet Infect Dis* 2002;**2**:494–501. doi:10.1016/S1473-3099(02)00347-X

3 World Health Organization. Leishmaniasis [online]. https://www.who.int/news-room/fact-sheets/detail/leishmaniasis (accessed 12 May 2023).

4 GBD 2019 Diseases and Injuries Collaborators. Global burden of 369 diseases and injuries in 204 countries and territories, 1990-2019: a systematic analysis for the Global Burden of Disease Study 2019. *Lancet* 2020;**396**:1204–22. doi:10.1016/S0140-6736(20)30925-9

5 Alves F, Bilbe G, Blesson S, *et al.* Recent development of visceral leishmaniasis treatments: Successes, pitfalls, and perspectives. *Clin Microbiol Rev* 2018;**31**:e00048-18. doi:10.1128/cmr.00048-18

6 Alvar J, Aparicio P, Aseffa A, *et al.* The relationship between leishmaniasis and AIDS: The second 10 years. *Clin Microbiol Rev* 2008;**21**:334–59. doi:10.1128/CMR.00061-07

7 Debray TPA, Riley RD, Rovers MM, *et al.* Individual Participant Data (IPD) Meta-analyses of Diagnostic and Prognostic Modeling Studies: Guidance on Their Use. *PLoS Med* 2015;**12**:e1001886. doi:10.1371/journal.pmed.1001886

For peer review only

8 Ministério da Saúde. Guia de Vigilância em Saúde. 5ª edição [online]. 2021. https://www.gov.br/saude/pt-br/centrais-de-conteudo/publicacoes/svsa/vigilancia/guia-de-vigilancia-em-saude_5ed_21nov21_isbn5.pdf (accessed 12 May 2023).

9 Naylor-Leyland G, Collin SM, Gatluak F, *et al.* The increasing incidence of visceral leishmaniasis relapse in South Sudan: A retrospective analysis of field patient data from 2001–2018. *PLoS Negl Trop Dis* 2022;**16**:e0010696. doi:10.1371/journal.pntd.0010696

10 Wynants L, Van Calster B, Collins GS, *et al.* Prediction models for diagnosis and prognosis of covid-19: Systematic review and critical appraisal. *BMJ* 2020;**369**:m1328. doi:10.1136/bmj.m1328

11 Peetluk LS, Ridolfi FM, Rebeiro PF, *et al.* Systematic review of prediction models for pulmonary tuberculosis treatment outcomes in adults. *BMJ Open* 2021;**11**:e044687. doi:10.1136/bmjopen-2020-044687

12 Njim T, Tanyitiku BS. Prognostic models for the clinical management of malaria and its complications: A systematic review. *BMJ Open* 2019;**9**:e030793. doi:10.1136/bmjopen-2019-030793

13 Altman DG, Vergouwe Y, Royston P, *et al.* Prognosis and prognostic research: Validating a prognostic model. *BMJ* 2009;**338**:b605. doi:10.1136/bmj.b605

14 Steyerberg EW, Moons KGM, van der Windt DA, *et al.* Prognosis Research Strategy (PROGRESS) 3: Prognostic Model Research. *PLoS Med* 2013;**10**:e1001381. doi:10.1371/journal.pmed.1001381

15 Wyatt JC, Altman DG. Commentary: Prognostic models: Clinically useful or quickly forgotten? *BMJ* 1995;**311**. doi:10.1136/bmj.311.7019.1539

16 Page MJ, McKenzie JE, Bossuyt PM, *et al.* The PRISMA 2020 statement: An updated guideline for reporting systematic reviews. *BMJ* 2021;**372**:n71. doi:10.1136/bmj.n71

17 Snell K, Levis B, Damen JAA, *et al.* Transparent reporting of multivariable prediction models for individual prognosis or diagnosis: checklist for systematic reviews and meta-analyses (TRIPOD-SRMA). *BMJ* 2023;**3**:e073538. doi:10.1136/bmj-2022-073538

18 Moher D, Shamseer L, Clarke M, *et al.* Preferred reporting items for systematic review and meta-analysis protocols (PRISMA-P) 2015 statement. *Syst Rev* 2015;**4**. doi:10.1186/2046-4053-4-1

19 Debray TPA, Damen JAAG, Snell KIE, *et al.* A guide to systematic review and meta-analysis of prediction model performance. *BMJ* 2017;**356**:i6460. doi:10.1136/bmj.i6460

20 Moons KGM, de Groot JAH, Bouwmeester W, *et al.* Critical Appraisal and Data Extraction for Systematic Reviews of Prediction Modelling Studies: The CHARMS Checklist. *PLoS Med* 2014;**11**:e1001744. doi:10.1371/journal.pmed.1001744

21 Collins GS, Reitsma JB, Altman DG, *et al.* Transparent reporting of a multivariable prediction model for individual prognosis or diagnosis (TRIPOD): The TRIPOD Statement. *Eur Urol* 2015;**67**:W1-73. doi:10.1016/j.eururo.2014.11.025

For peer review only

22 Moons KGM, Wolff RF, Riley RD, *et al.* PROBAST: A tool to assess risk of bias and applicability of prediction model studies: Explanation and elaboration. *Ann Intern Med* 2019;**170**:W1–33. doi:10.7326/M18-1377

23 Riley RD, Moons KGM, Snell KIE, *et al.* A guide to systematic review and meta-analysis of prognostic factor studies. *BMJ* 2019;**364**:k4597. doi:10.1136/bmj.k4597

24 Ingui BJ, Rogers MAM. Searching for clinical prediction rules in MEDLINE. *J Am Med Inform Assoc* 2001;**8**:391–7. doi:10.1136/jamia.2001.0080391

25 Geersing GJ, Bouwmeester W, Zuithoff P, *et al.* Search filters for finding prognostic and diagnostic prediction studies in medline to enhance systematic reviews. *PLoS One* 2012;**7**:e32844. doi:10.1371/journal.pone.0032844

26 Veritas Health Innovation. Covidence systematic review sofware. Covidence. 2019.

27 Harris PA, Taylor R, Minor BL, *et al.* The REDCap consortium: Building an international community of software platform partners. J Biomed Inform. 2019;**95**. doi:10.1016/j.jbi.2019.103208

28 Wolff R, Moons K, Riley R, *et al.* PROBAST (Prediction model study Risk Of Bias Assessment Tool). *Ann Intern Med* 2019;**170**:51–8. doi:10.7326/M18-1376

29 Sterne JAC, Savović J, Page MJ, *et al.* RoB 2: a revised tool for assessing risk of bias in randomised trials. *BMJ* 2019;**366**:l4898. doi:10.1136/bmj.l4898

30 Sterne JA, Hernán MA, Reeves BC, *et al.* ROBINS-I: A tool for assessing risk of bias in non-randomised studies of interventions. *BMJ* 2016;**355**:i4919. doi:10.1136/bmj.i4919

31 Damen JAA, Moons KGM, van Smeden M, *et al.* How to conduct a systematic review and meta-analysis of prognostic model studies. *Clin Microbiol Infect* 2022;**29**:434–40. doi:10.1016/j.cmi.2022.07.019

32 Debray TPA, Collins GS, Riley RD, *et al.* Transparent reporting of multivariable prediction models developed or validated using clustered data (TRIPOD-Cluster): explanation and elaboration. *BMJ* 2023;**380**:e071058. doi:10.1136/bmj-2022-071058

33 Bush JT, Wasunna M, Alves F, *et al.* Systematic review of clinical trials assessing the therapeutic efficacy of visceral leishmaniasis treatments: A first step to assess the feasibility of establishing an individual patient data sharing platform. *PLoS Negl Trop Dis* 2017;**11**:e0005781. doi:10.1371/journal.pntd.0005781

For peer review only

Search strategy

Tables 1-5 describe the search terms for each database (Ovid MEDLINE; Ovid Embase, the Web of Science Core Collection, SciELO and LILACS, respectively). All searches performed on 1st March 2023.

Query #	Query terms
1	Leishmaniasis, Visceral/
2	((Leishmaniasis and Visceral) or (Leishmania and infantum) or (Leishmania and donovani) or (Kala and azar)).ti,ab,kw.
3	1 or 2
4	Validat$.ti,ab. or Predict$.ti. or Rule$.ti,ab. or (Predict$ adj2 (Outcome$ or Risk$ or Model$)).ti,ab. or ((History or Variable$ or Criteria or Scor$ or Characteristic$ or Finding$ or Factor$) adj2 (Predict$ or Model$ or Decision$ or Identif$ or Prognos$)).ti,ab. or (Decision$ adj2 (Model$ or Clinical$)).ti,ab. or (Prognostic adj2 (History or Variable$ or Criteria or Scor$ or Characteristic$ or Finding$ or Factor$ or Model$)).ti,ab.
5	logistic models/
6	decision*.ti,ab
7	5 and 6
8	4 or 7
9	(Stratification or ROC Curve or Discrimination or Discriminate or c-statistic or c statistic or Area under the curve or AUC or Calibration or Indices or Algorithm or Multivariable).ti,ab.
10	roc curve/
11	8 or 9 or 10
12	3 and 11

Table 1: Search strategy - Ovid MEDLINE

**Query #**	**Query terms**
1	visceral leishmaniasis/
2	((Leishmaniasis and Visceral) or (Leishmania and infantum) or (Leishmania and donovani) or (Kala and azar)).ti,ab,kw.
3	1 or 2
4	Validat$.ti,ab. or Predict$.ti. or Rule$.ti,ab. or (Predict$ adj2 (Outcome$ or Risk$ or Model$)).ti,ab. or ((History or Variable$ or Criteria or Scor$ or Characteristic$ or Finding$ or Factor$) adj2 (Predict$ or Model$ or Decision$ or Identif$ or Prognos$)).ti,ab. or (Decision$ adj2 (Model$ or Clinical$)).ti,ab. or (Prognostic adj2 (History or Variable$ or Criteria or Scor$ or Characteristic$ or Finding$ or Factor$ or Model$)).ti,ab.
5	statistical model/
6	decision*.ti,ab.
7	5 and 6
8	4 or 7
9	(Stratification or ROC Curve or Discrimination or Discriminate or c-statistic or c statistic or Area under the curve or AUC or Calibration or Indices or Algorithm or Multivariable).ti,ab.
10	receiver operating characteristic/
11	8 or 9 or 10
12	3 and 11

Table 2: Search strategy - Ovid Embase

For peer review only

Query #	Query terms
1	TS=((Leishmaniasis and Visceral) or (Leishmania and infantum) or (Leishmania and donovani) or (Kala and azar))
2	TS=(Validat$ or Rule$ or (Predict$ near/2 (Outcome$ or Risk$ or Model$)) or ((History or Variable$ or Criteria or Scor$ or Characteristic$ or Finding$ or Factor$) near/2 (Predict$ or Model$ or Decision$ or Identif$ or Prognos$)) or (Decision$ near/2 (Model$ or Clinical$)) or (Prognostic near/2 (History or Variable$ or Criteria or Scor$ or Characteristic$ or Finding$ or Factor$ or Model$)))
3	TI=(Predict$)
4	TS=(Stratification or ROC Curve or Discrimination or Discriminate or c-statistic or c statistic or Area under the curve or AUC or Calibration or Indices or Algorithm or Multivariable)
5	#4 OR #3 OR #2
6	#5 AND #1

Table 3: Search strategy - Web of Science Core Collection

**Query #**	**Query terms**
1	All indexes: ((Leishmaniasis and Visceral) or (Leishmania and infantum) or (Leishmania and donovani) or (Kala and azar))
2	All indexes: Validat* or Rule* or (Predict* and (Outcome* or Risk* or Model*)) or ((History or Variable* or Criteria or Scor* or Characteristic* or Finding* or Factor*) and (Predict* or Model* or Decision* or Identif* or Prognos*)) or (Decision* and (Model* or Clinical*)) or (Prognostic and (History or Variable* or Criteria or Scor* or Characteristic* or Finding* or Factor* or Model*)) or Stratification or ROC Curve or Discrimination or Discriminate or c-statistic or c statistic or Area under the curve or AUC or Calibration or Indices or Algorithm or Multivariable
3	1 AND 2

Table 4: Search strategy – SciELO

Query #	Query terms
1	(tw:(((Leishmaniasis and Visceral) or (Leishmania and infantum) or (Leishmania and donovani) or (Kala and azar)))) AND (tw:(Validat* or Rule* or (Predict* and (Outcome* or Risk* or Model*)) or ((History or Variable* or Criteria or Scor* or Characteristic* or Finding* or Factor*) and (Predict* or Model* or Decision* or Identif* or Prognos*)) or (Decision* and (Model* or Clinical*)) or (Prognostic and (History or Variable* or Criteria or Scor* or Characteristic* or Finding* or Factor* or Model*)) or Stratification or ROC Curve or Discrimination or Discriminate or c-statistic or c statistic or Area under the curve or AUC or Calibration or Indices or Algorithm or Multivariable))

Table 5: Search strategy - LILACS

For peer review only - http://bmjopen.bmj.com/site/about/guidelines.xhtml

Reporting checklist for protocol of a systematic review and meta analysis.

Based on the PRISMA-P guidelines.

Instructions to authors

Complete this checklist by entering the page numbers from your manuscript where readers will find each of the items listed below.

Your article may not currently address all the items on the checklist. Please modify your text to include the missing information. If you are certain that an item does not apply, please write "n/a" and provide a short explanation.

Upload your completed checklist as an extra file when you submit to a journal.

In your methods section, say that you used the PRISMA-Preporting guidelines, and cite them as:

Moher D, Shamseer L, Clarke M, Ghersi D, Liberati A, Petticrew M, Shekelle P, Stewart LA. Preferred Reporting Items for Systematic Review and Meta-Analysis Protocols (PRISMA-P) 2015 statement. Syst Rev. 2015;4(1):1.

For peer review only

		Reporting Item	Page Number
**Title**			
Identification	#1a	Identify the report as a protocol of a systematic review	1
Update	#1b	If the protocol is for an update of a previous systematic review, identify as such	n/a

Registration

	#2	If registered, provide the name of the registry (such as PROSPERO) and registration number	1

Authors

Contact	#3a	Provide name, institutional affiliation, e-mail address of all protocol authors; provide physical mailing address of corresponding author	7
Contribution	#3b	Describe contributions of protocol authors and identify the guarantor of the review	8

Amendments

	#4	If the protocol represents an amendment of a previously completed or published protocol, identify as such and list changes; otherwise, state plan for documenting important protocol amendments	n/a

Support

Sources	#5a	Indicate sources of financial or other support for the review	8
Sponsor	#5b	Provide name for the review funder and / or sponsor	8
Role of sponsor or funder	#5c	Describe roles of funder(s), sponsor(s), and / or institution(s), if any, in developing the protocol	8

Introduction

For peer review only

Rationale	#6	Describe the rationale for the review in the context of what is already known	2,3
Objectives	#7	Provide an explicit statement of the question(s) the review will address with reference to participants, interventions, comparators, and outcomes (PICO)	2,3
**Methods**			
Eligibility criteria	#8	Specify the study characteristics (such as PICO, study design, setting, time frame) and report characteristics (such as years considered, language, publication status) to be used as criteria for eligibility for the review	3
Information sources	#9	Describe all intended information sources (such as electronic databases, contact with study authors, trial registers or other grey literature sources) with planned dates of coverage	4
Search strategy	#10	Present draft of search strategy to be used for at least one electronic database, including planned limits, such that it could be repeated	4, Suppl
Study records - data management	#11a	Describe the mechanism(s) that will be used to manage records and data throughout the review	4
Study records - selection process	#11b	State the process that will be used for selecting studies (such as two independent reviewers) through each phase of the review (that is, screening, eligibility and inclusion in meta-analysis)	4

For peer review only

Study records - data collection process	#11c	Describe planned method of extracting data from reports (such as piloting forms, done independently, in duplicate), any processes for obtaining and confirming data from investigators	4
Data items	#12	List and define all variables for which data will be sought (such as PICO items, funding sources), any pre-planned data assumptions and simplifications	5,6
Outcomes and prioritization	#13	List and define all outcomes for which data will be sought, including prioritization of main and additional outcomes, with rationale	3
Risk of bias in individual studies	#14	Describe anticipated methods for assessing risk of bias of individual studies, including whether this will be done at the outcome or study level, or both; state how this information will be used in data synthesis	6
Data synthesis	#15a	Describe criteria under which study data will be quantitatively synthesised	n/a
Data synthesis	#15b	If data are appropriate for quantitative synthesis, describe planned summary measures, methods of handling data and methods of combining data from studies, including any planned exploration of consistency (such as I2, Kendall's τ)	n/a
Data synthesis	#15c	Describe any proposed additional analyses (such as sensitivity or subgroup analyses, meta-regression)	n/a

For peer review only

Data synthesis	#15d	If quantitative synthesis is not appropriate, describe the type of summary planned	6
Meta-bias(es)	#16	Specify any planned assessment of meta-bias(es) (such as publication bias across studies, selective reporting within studies)	n/a
Confidence in cumulative evidence	#17	Describe how the strength of the body of evidence will be assessed (such as GRADE)	n/a

The PRISMA-P elaboration and explanation paper is distributed under the terms of the Creative Commons Attribution License CC-BY. This checklist was completed on 12. May 2023 using https://www.goodreports.org/, a tool made by the EQUATOR Network in collaboration with Penelope.ai

For peer review only

BMJ Open

Prognostic prediction models for clinical outcomes in patients diagnosed with visceral leishmaniasis: protocol for a systematic review

Journal:	*BMJ Open*
Manuscript ID	bmjopen-2023-075597.R1
Article Type:	Protocol
Date Submitted by the Author:	02-Oct-2023
Complete List of Authors:	Wilson, James; Infectious Diseases Data Observatory (IDDO); University of Oxford Centre for Tropical Medicine and Global Health<br>Chowdhury, Forhad; Infectious Diseases Data Observatory (IDDO); University of Oxford Centre for Tropical Medicine and Global Health<br>Hassan, Shermarke; Infectious Diseases Data Observatory (IDDO); University of Oxford Centre for Tropical Medicine and Global Health<br>Harriss, Elinor; Bodleian Health Care Libraries, University of Oxford<br>Alves, Fabiana; Drugs for Neglected Disease Initiative<br>Dahal, Prabin; Infectious Diseases Data Observatory (IDDO); University of Oxford Centre for Tropical Medicine and Global Health<br>Stepniewska, Kasia; Infectious Diseases Data Observatory (IDDO); University of Oxford Centre for Tropical Medicine and Global Health<br>Guérin, Philippe J.; Infectious Diseases Data Observatory (IDDO); University of Oxford Centre for Tropical Medicine and Global Health
**Primary Subject Heading**:	Infectious diseases
Secondary Subject Heading:	Research methods
Keywords:	Prognosis, Neglected Diseases, INFECTIOUS DISEASES, Systematic Review, STATISTICS & RESEARCH METHODS, Protocols & guidelines < HEALTH SERVICES ADMINISTRATION & MANAGEMENT

SCHOLARONE™
Manuscripts

Prognostic prediction models for clinical outcomes in patients diagnosed with visceral leishmaniasis: protocol for a systematic review

James P Wilson[1,2†], Forhad Chowdhury[1,2], Shermarke Hassan[1,2], Eli Harriss[3], Fabiana Alves[4], Prabin Dahal[1,2], Kasia Stepniewska[1,2], Philippe J Guérin[1,2]

[†]Correspondence to:
James P Wilson
james.wilson@iddo.org

For peer review only

ABSTRACT

Introduction

Visceral leishmaniasis (VL) is a neglected tropical disease responsible for many thousands of preventable deaths each year. Symptomatic patients often struggle to access effective treatment, without which death is the norm. Risk prediction tools support clinical teams and policymakers in identifying high-risk patients who could benefit from more intensive management pathways. Investigators interested in using their clinical data for prognostic research should first identify currently available models that are candidates for validation and possible updating. Addressing these needs, we aim to identify, summarise and appraise the available models predicting clinical outcomes in VL patients.

Methods and analysis

We will include studies that have developed, validated or updated prognostic models predicting future clinical outcomes in patients diagnosed with VL. Systematic reviews and meta-analyses that include eligible studies are also considered for review. Conference abstracts and educational theses are excluded. Data extraction, appraisal and reporting will follow current methodological guidelines. Ovid Embase; Ovid MEDLINE; the Web of Science Core Collection, SciELO and LILACS are searched from database inception to 1st March 2023 using terms developed for the identification of prediction models, and with no language restriction. Screening, data extraction, and risk of bias assessment will

be performed in duplicate with discordance resolved by a third independent reviewer. Risk of bias will be assessed using the PROBAST tool. Tables and figures will compare and contrast key model information, including source data, participants, model development and performance measures, and risk of bias. We will consider the strengths, limitations and clinical applicability of the identified models.

Ethics and dissemination

Ethics approval is not required for this review. The systematic review and all accompanying data will be submitted to an open-access journal. Findings will also be disseminated through the research group's website (www.iddo.org/research-themes/visceral-leishmaniasis) and social media channels.

Study registration

PROSPERO, CRD42023417226.

Keywords

Visceral Leishmaniasis; Review, Systematic; Clinical Decision Rules; Prognosis; Prognostic Factors; Neglected Tropical Diseases

Strengths and limitations of this study

- We present a protocol for a robust and comprehensive systematic review of visceral leishmaniasis (VL) prognostic models, using current best-practice guidelines on data extraction, risk of bias assessment and reporting.
- Inclusion criteria are designed to identify a broad range of VL prognostic model studies, including all patients with a VL diagnosis, and with no exclusions based on treatment setting, type of clinical outcome or prediction horizon.

For peer review only

- We describe a comprehensive and evidence-based search strategy to identify a broad range of prognostic model studies across five large bibliographic databases, with no limitations on language or initial publication date.
- Unpublished, non-peer-reviewed studies, such as conference abstracts and educational theses, are not included in the eligibility criteria.
- A systematic assessment of the current use and impact of VL prognostic models is considered outside the scope of the planned review.

INTRODUCTION

Visceral leishmaniasis (VL), a parasitic infection transmitted between mammalian hosts via the bite of an infected sand fly, is a disease mostly prevalent in tropical regions that disproportionately touches vulnerable people affected by forced migration, malnutrition and poverty.[1] The disease often presents insidiously with fever, splenomegaly and weight-loss, and is almost always fatal without effective treatment.[2] The World Health Organization (WHO) estimates an incidence of 50,000 to 90,000 cases per year,[3] resulting in approximately 400,000 disability-adjusted life years lost and over 5,000 deaths.[4] However, accurate estimates of disease burden are obfuscated by limited country-level reporting, evolving dense foci of infection in remote areas, and a paucity of active surveillance.[1] Despite progress made over the last 20 years, successful treatment remains challenged by high drug costs, prolonged treatment courses requiring hospitalisation and frequent drug side effects.[5] Patients with previous treatment failure or immunosuppressive comorbidities such as advanced human immunodeficiency virus (HIV) suffer from particularly high relapse and mortality rates.[1,6]

To optimise individual patient care and effectively balance the distribution of constrained resources, identification of patients at high risk of treatment failure and subsequent death is crucial. Risk stratification is also important on a population level; elimination programmes in endemic areas can use risk prediction tools to strategically target patients prone to treatment failure and hence reduce

For peer review only

the infectious reservoir driving onward transmission. Prognostic prediction models (referred henceforth as prognostic models) play a central role in VL risk stratification; informing healthcare providers, policymakers and patients on the treatment setting, treatment regimen and intensity of follow-up.[7–9]

Systematic reviews of prognostic models have been published across a range of infectious diseases,[10–12] serving not only to inform healthcare providers on available risk stratification tools, but also as a research tool to identify candidate models for external validation or updating (recalibration) with data from new settings. Indeed, the lack of external validation studies is considered the greatest barrier to the broader acceptance of prognostic models as a reliable and acceptable clinical tool.[13–15]

Aim

We will perform a systematic literature review to identify, summarise, and appraise prognostic models in patients diagnosed with VL. Specifically, we focus on models that predict clinical outcomes such as treatment failure (initial failure or relapse) and death, developed subsequent to VL diagnosis.

The review will serve two principal purposes:

(i) inform stakeholders, such as policymakers and healthcare workers directly involved in the treatment of VL patients, on the available prognostic models and their setting-specific clinical utility, strengths and limitations.

(ii) Inform researchers interested in using their own data for the development, validation or updating of VL prognostic models.

METHODS AND ANALYSIS

This systematic review will adhere to PRISMA (preferred reporting items for systematic reviews and meta-analyses), a reporting guideline for systematic reviews,[16] and TRIPOD-SRMA (transparent reporting of multivariable prediction models for individual prognosis or diagnosis: checklist for

For peer review only

systematic reviews and meta-analysis), a reporting guideline for systematic reviews of prediction models.[17]

This protocol is registered with PROSPERO (ID: CRD42023417226). Important protocol amendments will be documented with PROSPERO. We use the PRISMA-P checklist to guide reporting of the protocol (Supplemental Material 1).[18]

Study eligibility

We follow a PICOTS (Population, Index model, Comparator model, Outcomes, Timing, Setting) approach to frame our review question and inclusion criteria (Table 1).[19,20]

Our population of interest includes all patients with a confirmed or suspected diagnosis of VL as reported by the study authors. We include all published, peer-reviewed studies that develop, externally validate, update, or any combination thereof, a prognostic model with the intention of predicting individual clinical outcomes following VL diagnosis.

Table 1. *PICOTS approach to frame the research question*

**Population**	All patients with a confirmed or suspected diagnosis of visceral leishmaniasis as per study authors.
**Index model**	All published prognostic models that develop, validate and/or update (recalibrate/extend) a risk model.
**Comparator model**	Not applicable.
**Outcomes**	Any clinical outcome that occurs following diagnosis.
**Timing**	All prognostic models developed at the time of, or following diagnosis. No restriction on the prediction horizon.
**Setting**	No restriction.

In accordance with expert guidance on the methodology of prediction model research,[14,21,22] we define a prognostic model as a multivariable model (including two or more predictors) where the intention is to predict outcomes at the individual patient level. Prognostic model studies are

For peer review only

distinguished from predictor finding or prognostic factor studies, where the intent is to investigate the effect of a single or group of factors on an outcome of interest.[23] We therefore exclude all studies that present models where the aim is not to predict risk at the individual patient level. We also exclude unpublished and non-peer-reviewed studies, including conference abstracts and educational theses, studies that only report diagnostic prediction models, and animal studies.

To complement the systematic review, using the same search strategy we will identify (i) systematic reviews and meta-analyses of prognostic models, and (ii) any impact studies that investigate the clinical outcomes of using versus not using an eligible prognostic model. These studies will not be subject to formal data extraction, but will be summarised in a narrative review.

Search strategy

An information specialist (EH) created the search strategies to retrieve relevant records from the following databases: Ovid Embase; Ovid MEDLINE; the Web of Science Core Collection, SciELO and LILACS. The databases were searched from inception to 1st March 2023 with no language restriction. Where necessary, published in languages not spoken by the authors will be translated using the Google Translate service (http://translate.google.com), or otherwise using a professional translation service. The search strategy used text words and relevant indexing terms to retrieve studies describing eligible prognostic models. The Ingui search filter was combined with an additional search string developed by Geersing et al,[24,25] and adapted for Ovid Embase (Table 2) and remaining bibliographic databases (Supplemental Material 2).

Table 2. *Search strategy initially developed in Ovid Embase and searched on 1st March 2023 (search queries were subsequently adapted to other bibliographic databases)*

Query #	Query terms
1	visceral leishmaniasis/
2	((Leishmaniasis and Visceral) or (Leishmania and infantum) or (Leishmania and donovani) or (Kala and azar)).ti,ab,kw.

For peer review only

3	1 or 2
4	Validat$.ti,ab. or Predict$.ti. or Rule$.ti,ab. or (Predict$ adj2 (Outcome$ or Risk$ or Model$)).ti,ab. or ((History or Variable$ or Criteria or Scor$ or Characteristic$ or Finding$ or Factor$) adj2 (Predict$ or Model$ or Decision$ or Identif$ or Prognos$)).ti,ab. or (Decision$ adj2 (Model$ or Clinical$)).ti,ab. or (Prognostic adj2 (History or Variable$ or Criteria or Scor$ or Characteristic$ or Finding$ or Factor$ or Model$)).ti,ab.
5	statistical model/
6	decision*.ti,ab.
7	5 and 6
8	4 or 7
9	(Stratification or ROC Curve or Discrimination or Discriminate or c-statistic or c statistic or Area under the curve or AUC or Calibration or Indices or Algorithm or Multivariable).ti,ab.
10	receiver operating characteristic/
11	8 or 9 or 10
12	3 and 11

Selection process and data extraction

All references were exported to Covidence for deduplication and screening (Covidence systematic review software, Veritas Health Innovation, Melbourne, Australia. Available at www.covidence.org).[26] Google Scholar will be used as a grey literature source once screening is complete to identify additional relevant studies. Citation searching of all included studies will be performed to identify further studies for screening.

Studies identified from the search strategy are being independently screened by two reviewers (JW, FC). Preliminary screening is at title and abstract level followed by full-text screening. A third reviewer (PD), an experienced statistician, will make the final judgement on study inclusion if discordance remains following discussion between the two screening reviewers. A flow diagram will be presented as per the PRISMA 2020 checklist.[16]

For peer review only

Study information will be extracted, collected and managed using the REDCap electronic data capture tools hosted at the University of Oxford.[27] A data extraction form will be created based on the CHARMS (critical appraisal and data extraction for systematic reviews of prediction modelling studies) checklist for data extraction and PROBAST (prediction model risk of bias assessment tool; https://www.probast.org/) for assessing risk of bias (Table 3).[20,22] A pilot form will be trialled prior to data extraction. Two independent reviewers (JW, SH), both with expertise in prediction modelling, will independently extract the study information. Where discordance remains after discussion, a final decision will be made by a third expert reviewer (PD). Study authors will not be contacted to obtain information not reported in the study.

Table 3. *Information for data extraction and subsequent summary and appraisal*

Domain	Key Items
Source of data	Source of data (e.g., cohort, case-control, randomised trial participants, registry data, etc.)
Participants	Participant eligibility and recruitment method (e.g., location, number of centres, setting, inclusion and exclusion criteria)
	Participant description (age, sex, primary VL or relapse case, co-morbidities including HIV co-infection)
	Details of treatments received
	How VL diagnosis is defined (whether consistent for all participants, using serology and/or microscopy, molecular testing, clinical history and physical signs, etc)
	Study dates
Outcome(s) to be predicted	Type of outcome (e.g., single or combined endpoints)
	Definition and method for measurement of outcome (for example, is mortality disease-specific or all-cause, is cure/initial failure/relapse diagnosed based on clinical symptoms and/or diagnostic testing)
	Was the same outcome definition (and method for measurement) used in all patients?
	Time of outcome occurrence or summary of duration of follow-up
	Was the outcome assessed without knowledge of the candidate predictors (i.e., blinded)?

For peer review only

Candidate predictors	Number and type of predictors (e.g., demographics, patient history, physical examination, laboratory parameters, HIV status, disease characteristics, etc)
	Definition and method for measurement of candidate predictors (including whether defined and measured in a similar way for all participants)
	Timing of predictor measurement (e.g., at patient presentation, at diagnosis, at treatment initiation or otherwise)
	Handling of predictors in the modelling (e.g., continuous, linear, non-linear transformations or categorised)
Sample size	Number of participants and number of outcomes/events
	Events per candidate predictor
	Whether the authors describe a sample size calculation
Missing data	Number of participants with any missing value (including predictors and outcomes)
	Number of participants with missing data for each predictor
	Handling of missing data (e.g., complete-case analysis, imputation, or other methods)
Model development	Modelling method (e.g., logistic, survival, or other)
	Modelling assumptions satisfied
	Description of participants that were excluded from the analysis with justification
	Method for selection of predictors for inclusion in multivariable modelling (e.g., all candidate predictors, pre-selection based on unadjusted association with the outcome)
	Method for selection of predictors during multivariable modelling (e.g., full model approach, backward or forward selection) and criteria used (e.g., p-value, Akaike information criterion)
	Shrinkage of predictor weights or regression coefficients (e.g., no shrinkage, uniform shrinkage, penalized estimation)
Model performance	Calibration (calibration plot, calibration slope, Hosmer-Lemeshow test), discrimination (C-statistic, D-statistic, log-rank), and overall performance measures with confidence intervals
	Classification measures (e.g., sensitivity, specificity, predictive values, net reclassification improvement) and whether a priori cut points were used
Model evaluation	Method used for testing model performance: development dataset only (apparent performance, random split of data, resampling methods, e.g., bootstrap or cross-validation, none) or separate external validation

For peer review only

	For external validations; data source and participants to be described as per 'source of data' and 'participants' domains. Definitions and distributions (including missing data) of outcome and candidate predictors.
	In case of poor external validation, whether model was adjusted or updated (e.g., intercept recalibrated, predictor effects adjusted, or new predictors added)
Results	Final and other multivariable models presented, including predictor weights or regression coefficients, intercept, baseline survival, model performance measures (with standard errors or confidence intervals)
	Any alternative presentation of the final prediction models, e.g., sum score, nomogram, score chart, predictions for specific risk subgroups with performance
	Comparison of the definition and distribution of predictors (including missing data) for development and validation datasets
Interpretation and Discussion	Study authors' interpretation of presented models (intended use, clinical utility, etc)
	Study authors' reported strengths and limitations
Miscellaneous	Source of funding / sponsor
	Any declared conflicts of interest
	Methodological guidelines used

Adapted from the CHARMS Checklist and PROBAST risk of bias tool. VL: visceral leishmaniasis, HIV: human immunodeficiency virus.

For peer review only

Risk of bias

Risk of bias will be summarised and reported separately for each model development and external validation using PROBAST.[22] Two reviewers (JW, SH), will answer 20 signalling questions across four domains (participants, predictors, outcome and analysis), which will be used to judge the overall risk of bias as either 'low', 'high' or 'unclear'. Discordance between the two reviewers will be resolved by a third reviewer (PD).

If impact studies are identified, their risk of bias will be assessed using the Cochrane Risk of Bias tool for randomised comparative designs or the ROBINS-I (the risk of bias in non-randomised studies of interventions) tool for studies using a non-randomised comparative design.[28,29]

Data synthesis

We will present a narrative synthesis of our review findings. Key extracted information will be summarised in tabular form. Performance statistics, including measures of calibration and discrimination, where available, will be presented alongside their derivation method.

Figures will be used to concisely communicate important information, including (i) summary of the candidate and final predictors included in each model, and (ii) risk of bias assessment across the four domains (participants, predictors, outcomes and analysis).

Strengths and limitations of the identified models will be considered.

Given our aim is not to compare multiple external validations of a single model, we will not be performing a meta-analysis.[30]

Patient and public involvement

None.

ETHICS AND DISSEMINATION

Ethics approval is not required for this review. The systematic review will be submitted to an open-access journal for peer review and publication. Findings will also be disseminated through conference presentations, the research group's website (www.iddo.org/research-themes/visceral-leishmaniasis) and social media channels. All extracted information will be made freely available as supplemental material submitted during publication.

For peer review only

DISCUSSION

We present a protocol for the first systematic review of prognostic models for clinical outcomes in patients diagnosed with VL; a neglected tropical disease that affects some of the most disadvantaged communities in the world. Thoughtful risk stratification of patients using prognostic models can assist clinical decision making and inform policy, guiding the optimal allocation of often-limited resources. By identifying, summarising and appraising the published VL prognostic models, we hope that the planned systematic review will serve as a comprehensive resource for VL stakeholders, including healthcare workers, policymakers and researchers.

Clinical outcomes in VL are heterogeneous, with rates of initial treatment failure, relapse and mortality varying according to known and unknown factors. Many predictors of poor clinical outcomes have been identified, including extremes of patient age, severity of clinical signs and symptoms, laboratory investigations, the immune status of the patient (including the presence of advanced HIV), the patient's clinical management, geographical location, parasite genotype and resistance profile.[9,31,32] The relative contributions to patient outcomes of these inter-related factors can be described through multivariable modelling and subsequently used to estimate individual patient risk in the form of a prognostic model. However, such a model's performance in a new population can only be directly assessed through external validation. Indeed, this is considered an essential step prior to model use, but infrequently performed in practice.[21] The planned systematic review will concisely summarise key information presented across all identified prognostic model studies. VL healthcare providers and policymakers can then use this information, including performance estimates from external validations, to assess a model's applicability to their own patient population.

This review will not only serve healthcare providers and policymakers in identifying relevant risk stratification tools, but also provide a resource for research groups aiming to validate or update existing prognostic models. The Infectious Diseases Data Observatory (IDDO) is developing a data

For peer review only

repository of individual participant data (IPD) from VL clinical trials and observational studies (www.iddo.org/research-themes/visceral-leishmaniasis).[33] A VL data platform including IPD presents an exciting opportunity for the development, validation and updating of prognostic models.[34]

An important strength of the planned review is its broad eligibility criteria; we include models describing all clinical outcomes and impose no restriction on model setting, publication language, or prediction horizon (elapsed period between the intended time of model use and the outcome being predicted). Given concerns about study quality and accessibility, we will not be reviewing unpublished nor non-peer-previewed studies, such as conference abstracts or educational theses. A further limitation of the planned review is that we will not be contacting study authors to request unreported information, although we will explicitly report where information is missing. A systematic assessment of the current use and impact of VL prognostic models, including policy guidelines, is considered beyond the scope of the review, however, the review's findings will be considered in the context of current practice as understood by the authors.

In summary, we present a protocol for the systematic review of prognostic models of clinical outcomes for patients diagnosed with VL. With the aim of identifying, summarising and appraising the available risk models, we hope to provide a current reference to stakeholders engaged in VL patient care, policy and research.

Author affiliations

[1]Infectious Diseases Data Observatory (IDDO), Oxford, UK

[2]Centre for Tropical Medicine and Global Health, Nuffield Department of Medicine, University of Oxford, Oxford, UK

[3]Bodleian Health Care Libraries, University of Oxford, Oxford, UK

[4]Drugs for Neglected Diseases initiative, Geneva, Switzerland

[†]Corresponding author

For peer review only

Twitter

The Infectious Diseases Data Observatory: @IDDOnews

James P Wilson: @jamesemaj

Emails

James P Wilson: james.wilson@iddo.org

Forhad Chowdhury: forhad.chowdhury@ndm.ox.ac.uk

Shermarke Hassan: shermarke.hassan@iddo.org

Eli Harriss: eli.harriss@bodleian.ox.ac.uk

Fabiana Alves: falves@dndi.org

Prabin Dahal: prabin.dahal@iddo.org

Kasia Stepniewska: kasia.stepniewska@iddo.org

Philippe J Guérin: philippe.guerin@iddo.org

Contributors

The study concept and design were conceived by JW, PG, KS and PD. EH developed the search strategy. JW, FC and PD will complete the literature screening. JW, SH and PD will perform data extraction. KS and PD have provided statistical support. FA provided disease-specific expert advice. The first draft of this manuscript was prepared by JW, who will also draft the completed systematic review. All authors critically reviewed and approved the submitted version.

Funding

JW is undertaking a PhD supported in part by the University of Oxford through the Clarendon Scholarship and Clinician-Investigator Award and the Infectious Diseases Data Observatory, which is itself supported by a diverse portfolio of grants, including from the Bill & Melinda Gates Foundation (recipient: Prof. Philippe Guerin; ref: INV-004713). The funders have no role in the conception, design or conduct of the systematic review.

Competing interests

For peer review only

The authors declare no competing interests.

REFERENCES

1 Burza S, Croft SL, Boelaert M. Leishmaniasis. *The Lancet*. 2018;392:951–70.

2 Guerin PJ, Olliaro P, Sundar S, *et al.* Visceral leishmaniasis: current status of control, diagnosis, and treatment, and a proposed research and development agenda. *Lancet Infect Dis*. 2002;2:494–501.

3 World Health Organization. Leishmaniasis. https://www.who.int/news-room/fact-sheets/detail/leishmaniasis (accessed 24 August 2023)

4 Vos T, Lim SS, Abbafati C, *et al.* Global burden of 369 diseases and injuries in 204 countries and territories, 1990–2019: a systematic analysis for the Global Burden of Disease Study 2019. *The Lancet*. 2020;396:1204–22.

5 Alves F, Bilbe G, Blesson S, *et al.* Recent Development of Visceral Leishmaniasis Treatments: Successes, Pitfalls, and Perspectives. *Clin Microbiol Rev*. 2018;31:e00048-18.

6 Alvar J, Aparicio P, Aseffa A, *et al.* The Relationship between Leishmaniasis and AIDS: the Second 10 Years. *Clin Microbiol Rev*. 2008;21:334–59.

7 Ministry of Health (Brazil). *Guide to Health Surveillance. 5th Edition, revised and updated. (Guia de Vigilância em Saúde. 5ª edição revisada e atualizada.)*. Brasilia, Brazil . https://bvsms.saude.gov.br/bvs/publicacoes/guia_vigilancia_saude_5ed_rev_atual.pdf (accessed 24 August 2023)

8 Naylor-Leyland G, Collin SM, Gatluak F, *et al.* The increasing incidence of visceral leishmaniasis relapse in South Sudan: A retrospective analysis of field patient data from 2001–2018. *PLoS Negl Trop Dis*. 2022;16:e0010696.

9 Belo VS, Struchiner CJ, Barbosa DS, *et al.* Risk Factors for Adverse Prognosis and Death in American Visceral Leishmaniasis: A Meta-analysis. *PLoS Negl Trop Dis*. 2014;8:e2982.

10 Wynants L, Van Calster B, Collins GS, *et al.* Prediction models for diagnosis and prognosis of covid-19: systematic review and critical appraisal. *BMJ*. 2020;m1328.

11 Peetluk LS, Ridolfi FM, Rebeiro PF, *et al.* Systematic review of prediction models for pulmonary tuberculosis treatment outcomes in adults. *BMJ Open*. 2021;11:e044687.

12 Njim T, Tanyitiku BS. Prognostic models for the clinical management of malaria and its complications: a systematic review. *BMJ Open*. 2019;9:e030793.

13 Altman DG, Vergouwe Y, Royston P, *et al.* Prognosis and prognostic research: validating a prognostic model. *BMJ*. 2009;338:b605–b605.

14 Steyerberg EW, Moons KGM, Van Der Windt DA, *et al.* Prognosis Research Strategy (PROGRESS) 3: Prognostic Model Research. *PLoS Med*. 2013;10:e1001381.

For peer review only

15 Wyatt JC, Altman DG. Commentary: Prognostic models: clinically useful or quickly forgotten? *BMJ*. 1995;311:1539–41.

16 Page MJ, McKenzie JE, Bossuyt PM, *et al.* The PRISMA 2020 statement: an updated guideline for reporting systematic reviews. *BMJ*. 2021;n71.

17 Snell KIE, Levis B, Damen JAA, *et al.* Transparent reporting of multivariable prediction models for individual prognosis or diagnosis: checklist for systematic reviews and meta-analyses (TRIPOD-SRMA). *BMJ*. 2023;e073538.

18 PRISMA-P Group, Moher D, Shamseer L, *et al.* Preferred reporting items for systematic review and meta-analysis protocols (PRISMA-P) 2015 statement. *Syst Rev*. 2015;4:1.

19 Debray TPA, Damen JAAG, Snell KIE, *et al.* A guide to systematic review and meta-analysis of prediction model performance. *BMJ*. 2017;i6460.

20 Moons KGM, De Groot JAH, Bouwmeester W, *et al.* Critical Appraisal and Data Extraction for Systematic Reviews of Prediction Modelling Studies: The CHARMS Checklist. *PLoS Med*. 2014;11:e1001744.

21 Collins GS, Reitsma JB, Altman DG, *et al.* Transparent Reporting of a Multivariable Prediction Model for Individual Prognosis or Diagnosis (TRIPOD): The TRIPOD Statement. *Eur Urol*. 2015;67:1142–51.

22 Moons KGM, Wolff RF, Riley RD, *et al.* PROBAST: A Tool to Assess Risk of Bias and Applicability of Prediction Model Studies: Explanation and Elaboration. *Ann Intern Med*. 2019;170:W1.

23 Riley RD, Moons KGM, Snell KIE, *et al.* A guide to systematic review and meta-analysis of prognostic factor studies. *BMJ*. 2019;k4597.

24 Ingui BJ, Rogers MAM. Searching for Clinical Prediction Rules in MEDLINE. *J Am Med Inform Assoc*. 2001;8:391–7.

25 Geersing G-J, Bouwmeester W, Zuithoff P, *et al.* Search Filters for Finding Prognostic and Diagnostic Prediction Studies in Medline to Enhance Systematic Reviews. *PLoS ONE*. 2012;7:e32844.

26 Covidence systematic review software, Veritas Health Innovation, Melbourne, Australia.

27 Harris PA, Taylor R, Minor BL, *et al.* The REDCap consortium: Building an international community of software platform partners. *J Biomed Inform*. 2019;95:103208.

28 Sterne JA, Hernán MA, Reeves BC, *et al.* ROBINS-I: a tool for assessing risk of bias in non-randomised studies of interventions. *BMJ*. 2016;i4919.

29 Sterne JAC, Savović J, Page MJ, *et al.* RoB 2: a revised tool for assessing risk of bias in randomised trials. *BMJ*. 2019;l4898.

30 Damen JAA, Moons KGM, Van Smeden M, *et al.* How to conduct a systematic review and meta-analysis of prognostic model studies. *Clin Microbiol Infect*. 2023;29:434–40.

31 Grace CA, Sousa Carvalho KS, Sousa Lima MI, *et al.* Parasite Genotype Is a Major Predictor of Mortality from Visceral Leishmaniasis. *mBio*. 2022;13:e02068-22.

For peer review only

32 Collin S, Davidson R, Ritmeijer K, *et al.* Conflict and Kala-Azar: Determinants of Adverse Outcomes of Kala-Azar among Patients in Southern Sudan. *Clin Infect Dis*. 2004;38:612–9.

33 Bush JT, Wasunna M, Alves F, *et al.* Systematic review of clinical trials assessing the therapeutic efficacy of visceral leishmaniasis treatments: A first step to assess the feasibility of establishing an individual patient data sharing platform. *PLoS Negl Trop Dis*. 2017;11:e0005781.

34 Debray TPA, Riley RD, Rovers MM, *et al.* Individual Participant Data (IPD) Meta-analyses of Diagnostic and Prognostic Modeling Studies: Guidance on Their Use. *PLOS Med*. 2015;12:e1001886.

For peer review only

Reporting checklist for protocol of a systematic review and meta analysis.

Based on the PRISMA-P guidelines.

Instructions to authors

Complete this checklist by entering the page numbers from your manuscript where readers will find each of the items listed below.

Your article may not currently address all the items on the checklist. Please modify your text to include the missing information. If you are certain that an item does not apply, please write "n/a" and provide a short explanation.

Upload your completed checklist as an extra file when you submit to a journal.

In your methods section, say that you used the PRISMA-Preporting guidelines, and cite them as:

Moher D, Shamseer L, Clarke M, Ghersi D, Liberati A, Petticrew M, Shekelle P, Stewart LA. Preferred Reporting Items for Systematic Review and Meta-Analysis Protocols (PRISMA-P) 2015 statement. Syst Rev. 2015;4(1):1.

		Reporting Item	Page Number
**Title**			
Identification	#1a	Identify the report as a protocol of a systematic review	1
Update	#1b	If the protocol is for an update of a previous systematic review, identify as such	n/a
**Registration**			
	#2	If registered, provide the name of the registry (such as PROSPERO) and registration number	2
**Authors**			
Contact	#3a	Provide name, institutional affiliation, e-mail address of all protocol authors; provide physical mailing address of corresponding author	13-14

For peer review only

Contribution	#3b	Describe contributions of protocol authors and identify the guarantor of the review	14
**Amendments**			
	#4	If the protocol represents an amendment of a previously completed or published protocol, identify as such and list changes; otherwise, state plan for documenting important protocol amendments	5
**Support**			
Sources	#5a	Indicate sources of financial or other support for the review	14
Sponsor	#5b	Provide name for the review funder and / or sponsor	14
Role of sponsor or funder	#5c	Describe roles of funder(s), sponsor(s), and / or institution(s), if any, in developing the protocol	14
**Introduction**			
Rationale	#6	Describe the rationale for the review in the context of what is already known	3
Objectives	#7	Provide an explicit statement of the question(s) the review will address with reference to participants, interventions, comparators, and outcomes (PICO)	4
**Methods**			
Eligibility criteria	#8	Specify the study characteristics (such as PICO, study design, setting, time frame) and report characteristics (such as years considered, language, publication status) to be used as criteria for eligibility for the review	5
Information sources	#9	Describe all intended information sources (such as electronic databases, contact with study authors, trial registers or other grey literature sources) with planned dates of coverage	6
Search strategy	#10	Present draft of search strategy to be used for at least one electronic database, including planned limits, such that it could be repeated	6-7

For peer review only

Study records - data management	#11a	Describe the mechanism(s) that will be used to manage records and data throughout the review	7-8
Study records - selection process	#11b	State the process that will be used for selecting studies (such as two independent reviewers) through each phase of the review (that is, screening, eligibility and inclusion in meta-analysis)	7
Study records - data collection process	#11c	Describe planned method of extracting data from reports (such as piloting forms, done independently, in duplicate), any processes for obtaining and confirming data from investigators	8
Data items	#12	List and define all variables for which data will be sought (such as PICO items, funding sources), any pre-planned data assumptions and simplifications	8-10
Outcomes and prioritization	#13	List and define all outcomes for which data will be sought, including prioritization of main and additional outcomes, with rationale	8-10
Risk of bias in individual studies	#14	Describe anticipated methods for assessing risk of bias of individual studies, including whether this will be done at the outcome or study level, or both; state how this information will be used in data synthesis	10
Data synthesis	#15a	Describe criteria under which study data will be quantitatively synthesised	11
Data synthesis	#15b	If data are appropriate for quantitative synthesis, describe planned summary measures, methods of handling data and methods of combining data from studies, including any planned exploration of consistency (such as I2, Kendall's τ)	n/a
Data synthesis	#15c	Describe any proposed additional analyses (such as sensitivity or subgroup analyses, meta-regression)	n/a
Data synthesis	#15d	If quantitative synthesis is not appropriate, describe the type of summary planned	11
Meta-bias(es)	#16	Specify any planned assessment of meta-bias(es) (such as publication bias across studies, selective reporting within studies)	n/a

For peer review only

Confidence in cumulative evidence	#17	Describe how the strength of the body of evidence will be assessed (such as GRADE)	11

None The PRISMA-P elaboration and explanation paper is distributed under the terms of the Creative Commons Attribution License CC-BY. This checklist can be completed online using https://www.goodreports.org/, a tool made by the EQUATOR Network in collaboration with Penelope.ai

For peer review only

Supplemental Material - Search strategy

Tables 1-5 describe the search terms for each database (Ovid MEDLINE; Ovid Embase, the Web of Science Core Collection, SciELO and LILACS, respectively). All searches performed on 1st March 2023. No limit on publication date (from database inception to 1st March 2023).

Subsequent Google scholar grey literature review: ("visceral leishmaniasis" OR "Kala-azar") AND ("model" OR "prediction" OR "score" OR "prognostic"), run on July 17th 2023. The first 200 results were reviewed for relevance.

Query #	Query terms
1	Leishmaniasis, Visceral/
2	((Leishmaniasis and Visceral) or (Leishmania and infantum) or (Leishmania and donovani) or (Kala and azar)).ti,ab,kw.
3	1 or 2
4	Validat$.ti,ab. or Predict$.ti. or Rule$.ti,ab. or (Predict$ adj2 (Outcome$ or Risk$ or Model$)).ti,ab. or ((History or Variable$ or Criteria or Scor$ or Characteristic$ or Finding$ or Factor$) adj2 (Predict$ or Model$ or Decision$ or Identif$ or Prognos$)).ti,ab. or (Decision$ adj2 (Model$ or Clinical$)).ti,ab. or (Prognostic adj2 (History or Variable$ or Criteria or Scor$ or Characteristic$ or Finding$ or Factor$ or Model$)).ti,ab.
5	logistic models/
6	decision*.ti,ab
7	5 and 6
8	4 or 7
9	(Stratification or ROC Curve or Discrimination or Discriminate or c-statistic or c statistic or Area under the curve or AUC or Calibration or Indices or Algorithm or Multivariable).ti,ab.
10	roc curve/
11	8 or 9 or 10
12	3 and 11

Table 1: Search strategy - Ovid MEDLINE

**Query #**	**Query terms**
1	visceral leishmaniasis/
2	((Leishmaniasis and Visceral) or (Leishmania and infantum) or (Leishmania and donovani) or (Kala and azar)).ti,ab,kw.
3	1 or 2

For peer review only

4	Validat$.ti,ab. or Predict$.ti. or Rule$.ti,ab. or (Predict$ adj2 (Outcome$ or Risk$ or Model$)).ti,ab. or ((History or Variable$ or Criteria or Scor$ or Characteristic$ or Finding$ or Factor$) adj2 (Predict$ or Model$ or Decision$ or Identif$ or Prognos$)).ti,ab. or (Decision$ adj2 (Model$ or Clinical$)).ti,ab. or (Prognostic adj2 (History or Variable$ or Criteria or Scor$ or Characteristic$ or Finding$ or Factor$ or Model$)).ti,ab.
5	statistical model/
6	decision*.ti,ab.
7	5 and 6
8	4 or 7
9	(Stratification or ROC Curve or Discrimination or Discriminate or c-statistic or c statistic or Area under the curve or AUC or Calibration or Indices or Algorithm or Multivariable).ti,ab.
10	receiver operating characteristic/
11	8 or 9 or 10
12	3 and 11

Table 2: Search strategy - Ovid Embase

Query #	Query terms
1	TS=((Leishmaniasis and Visceral) or (Leishmania and infantum) or (Leishmania and donovani) or (Kala and azar))
2	TS=(Validat$ or Rule$ or (Predict$ near/2 (Outcome$ or Risk$ or Model$)) or ((History or Variable$ or Criteria or Scor$ or Characteristic$ or Finding$ or Factor$) near/2 (Predict$ or Model$ or Decision$ or Identif$ or Prognos$)) or (Decision$ near/2 (Model$ or Clinical$)) or (Prognostic near/2 (History or Variable$ or Criteria or Scor$ or Characteristic$ or Finding$ or Factor$ or Model$)))
3	TI=(Predict$)
4	TS=(Stratification or ROC Curve or Discrimination or Discriminate or c-statistic or c statistic or Area under the curve or AUC or Calibration or Indices or Algorithm or Multivariable)
5	#4 OR #3 OR #2
6	#5 AND #1

Table 3: Search strategy - Web of Science Core Collection

**Query #**	**Query terms**
1	All indexes: ((Leishmaniasis and Visceral) or (Leishmania and infantum) or (Leishmania and donovani) or (Kala and azar))

For peer review only

2	All indexes: Validat* or Rule* or (Predict* and (Outcome* or Risk* or Model*)) or ((History or Variable* or Criteria or Scor* or Characteristic* or Finding* or Factor*) and (Predict* or Model* or Decision* or Identif* or Prognos*)) or (Decision* and (Model* or Clinical*)) or (Prognostic and (History or Variable* or Criteria or Scor* or Characteristic* or Finding* or Factor* or Model*)) or Stratification or ROC Curve or Discrimination or Discriminate or c-statistic or c statistic or Area under the curve or AUC or Calibration or Indices or Algorithm or Multivariable
3	1 AND 2

Table 4: Search strategy – SciELO

Query #	Query terms
1	(tw:(((Leishmaniasis and Visceral) or (Leishmania and infantum) or (Leishmania and donovani) or (Kala and azar)))) AND (tw:(Validat* or Rule* or (Predict* and (Outcome* or Risk* or Model*)) or ((History or Variable* or Criteria or Scor* or Characteristic* or Finding* or Factor*) and (Predict* or Model* or Decision* or Identif* or Prognos*)) or (Decision* and (Model* or Clinical*)) or (Prognostic and (History or Variable* or Criteria or Scor* or Characteristic* or Finding* or Factor* or Model*)) or Stratification or ROC Curve or Discrimination or Discriminate or c-statistic or c statistic or Area under the curve or AUC or Calibration or Indices or Algorithm or Multivariable))

Table 5: Search strategy - LILACS

For peer review only
